# Retroperitoneal Ewing Sarcoma Masquerading as an Intraabdominal Abscess in a Teenager: A Rare Diagnostic Challenge

**DOI:** 10.7759/cureus.80853

**Published:** 2025-03-19

**Authors:** Daniel Hahn, Lauren Velasquez, Nickolas Muller, Margaret Lawless

**Affiliations:** 1 Internal Medicine, Touro College of Osteopathic Medicine, New York, USA; 2 Pathology, St. Mary's General Hospital, Passaic, USA

**Keywords:** chemotherapy, ewsr1 gene rearrangement, extraosseous ewing sarcoma, immunohistochemistry, retroperitoneal ewing sarcoma

## Abstract

Retroperitoneal Ewing sarcoma is a rare and aggressive extraskeletal variant of Ewing sarcoma, often presenting with nonspecific symptoms that can delay diagnosis. We discuss the case of a 19-year-old male who presented to the emergency room with progressive left abdominal and flank pain. Imaging revealed a retroperitoneal, necrotic mass and biopsy with immunohistochemistry confirmed retroperitoneal Ewing sarcoma with NKX2.2 and CD99 positivity. Fluorescence in situ hybridization (FISH) analysis showed that 98% of cells had an EWSR1 gene rearrangement. For treatment, the patient underwent a CT-guided biopsy and chemotherapy was initiated.

The sometimes-insidious progression of retroperitoneal Ewing sarcoma, coupled with the retroperitoneal space’s ability to accommodate tumors without significant symptoms, often leads to late-stage diagnosis and poor prognosis. This report emphasizes the importance of early recognition and highlights the need for further research into optimal diagnostic protocols and treatment strategies.

## Introduction

Ewing sarcomas are malignant tumors that most commonly arise in long bones but can present in soft tissue in rare cases [1]. A study involving a large database analysis of 1631 cases reported that the most common locations of Ewing sarcoma are in the axial skeleton (44.1%) and appendicular skeleton (37.5%) [2]. Extraskeletal Ewing sarcoma was less common, presenting in approximately 18.4% of all patients [2]. Retroperitoneal Ewing sarcoma is a rare variant of extraskeletal Ewing sarcoma, which has been reported in a limited number of individual case reports. It can be difficult to clinically diagnose due to its often delayed and nonspecific presentation. Patients usually present with complaints when the tumor becomes large enough to compress neighboring tissues, and these include abdominal pain, distension, nausea, and vomiting [3]. In this report, we describe the case of a 19-year-old male with retroperitoneal Ewing sarcoma who presented with symptoms and radiographic findings mimicking an intraabdominal abscess.

## Case presentation

A 19-year-old male presented with left lower quadrant abdominal pain and left flank pain for the past 48 hours. The pain was characterized as aching, aggravated by walking, relieved by lying still, non-radiating, and increased in intensity until it became debilitating. The patient denied fever, nausea, diarrhea, hematuria, and weight loss. He also denied any history of mechanical trauma. Due to the retroperitoneal location, an initial working diagnosis of intraabdominal abscess was entertained. Empiric antibiotic therapy was initiated, but the patient's symptoms persisted. 

**Figure 1:**
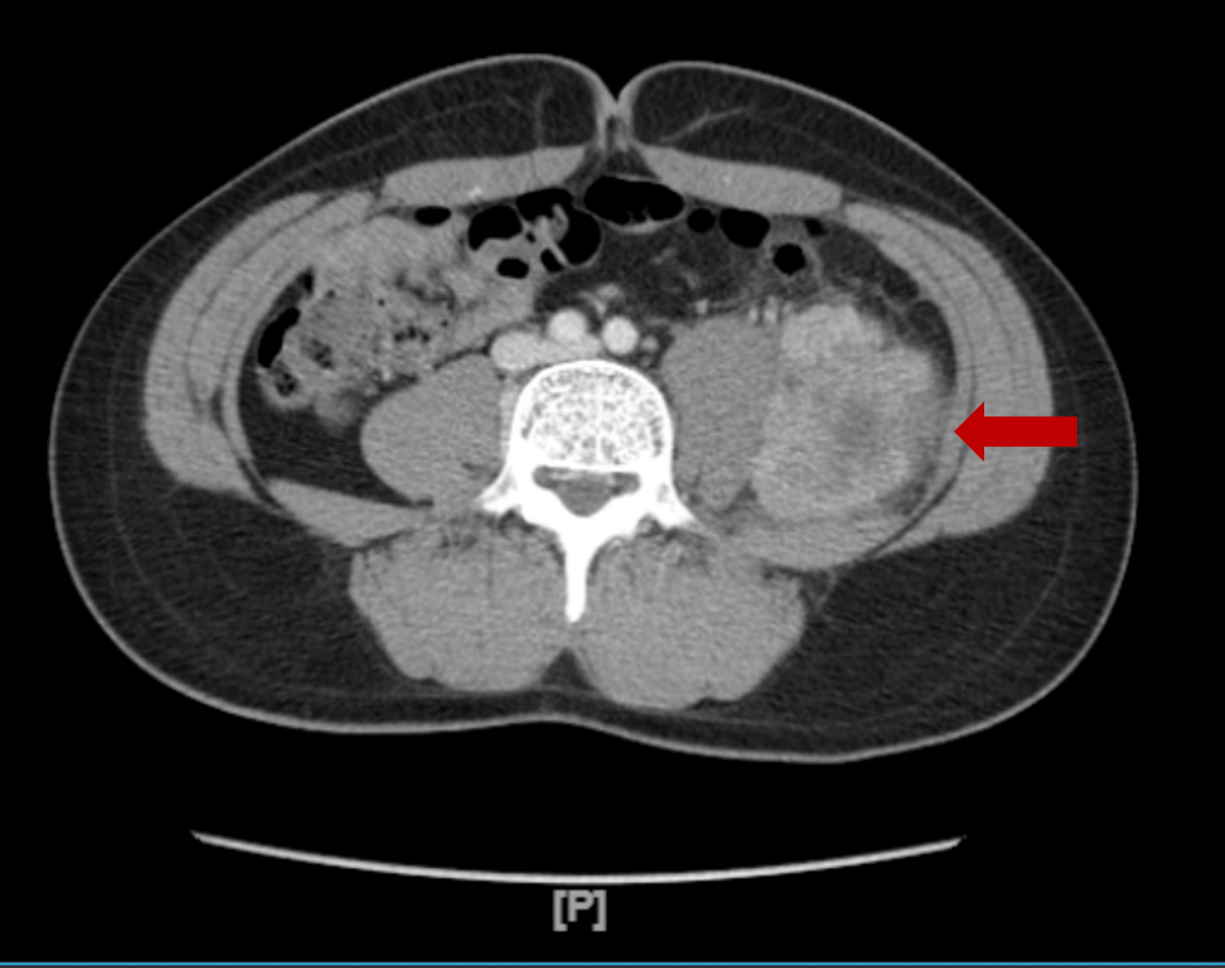
Abdominal CT scan with IV contrast showing retroperitoneal Ewing sarcoma (red arrow) CT: computed tomography; IV: intravenous

A CT scan of the abdomen showed an ovoid 6.1 x 4.8 x 5.6 cm retroperitoneal mass in the left iliac fossa (Figure 1). The mass was hypervascular with areas of relative central non-enhancement, suggestive of necrosis. There was no discrete abscess cavity. The left iliac muscle showed thickening and displacement of the left psoas muscle. The mass was mostly isodense relative to muscle, and there was infiltration into the adjacent fat. There was no evidence of additional osseous lesions, lymphadenopathy, bone destruction, or metastatic lesions.

**Figure 2:**
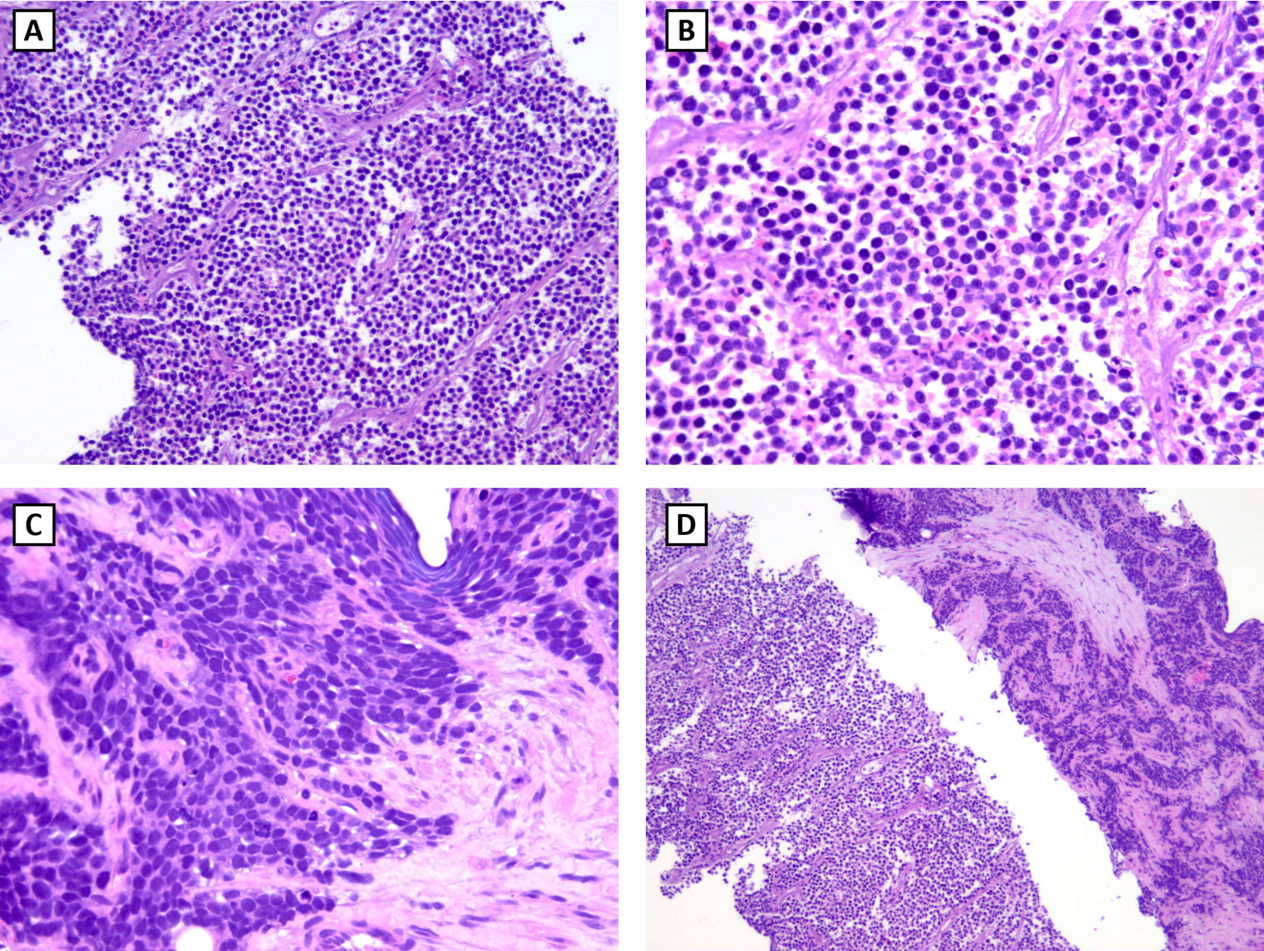
Hematoxylin and eosin (H&E) stain of retroperitoneal Ewing sarcoma A: small, blue, round cell tumor (50x); B: round-to-oval nuclei with a fine or stippled chromatin distribution (200x); C: cells with variability in sizes and molding (200x); D: internal fibrous septa (40x)

The patient underwent a biopsy with interventional radiology, but aspiration was unsuccessful. Core samples were obtained for analysis. The mass was composed of friable, gray-tan tissue. The biopsy showed an extensively necrotic small blue round cell tumor with dimorphic cellular morphology. Some classic morphological features were present, such as small cells that exhibited round-to-oval nuclei with a fine or stippled chromatin distribution (Figures 2A, 2B). The eosinophilic cytoplasm was scant with indistinct membranes. Some sections showed more variability in sizes and molding, creating a crowded appearance (Figure 2C). Fibrous septa could be seen internally and on the periphery (Figures 2B, 2D).

**Figure 3:**
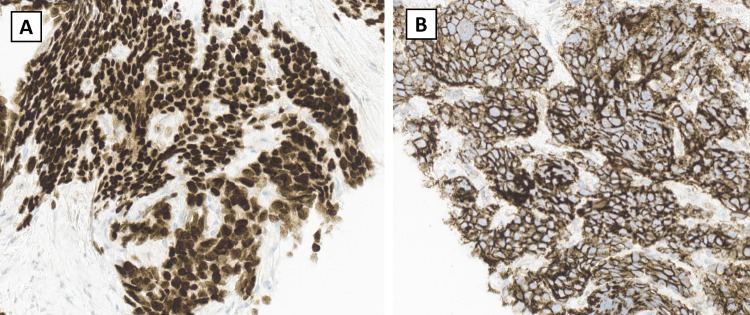
Immunohistochemistry stains of retroperitoneal Ewing sarcoma A: NKX2.2 stain with strong diffuse positivity and nuclear immunoreactivity; B: CD99 stain with strong, diffuse membranous pattern

Immunohistochemistry showed tumor cells with a strong, diffuse positivity and nuclear immunoreactivity for NKX2.2 (Figure 3A). Furthermore, CD99 immunohistochemistry staining revealed a strong, diffuse membranous pattern (Figure 3B). The tumor cells were negative for AE1/AE3, CD43, CD3, PAX5, CD34, and TdT.

**Figure 4:**
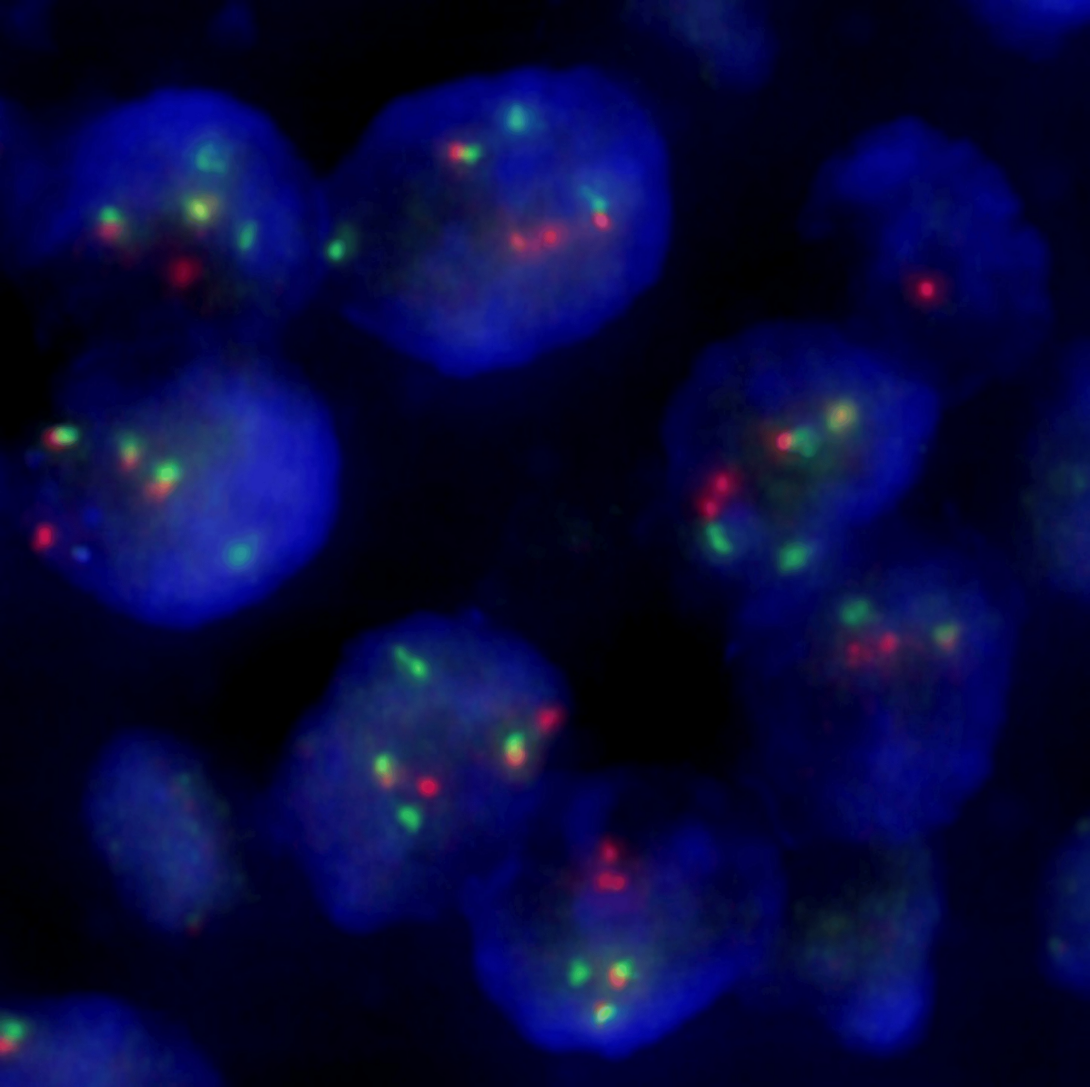
FISH analysis for EWSR1 break-apart 5’ in orange/red, 3’ in green FISH: fluorescence in situ hybridization

A fluorescence in situ hybridization (FISH) analysis detected EWSR1 gene rearrangement in 98% of the cells (Figure 4).

Shortly after consulting with the patient, a totally implantable venous access device (TIVAD) was placed with plans to administer cyclophosphamide and topotecan as initial chemotherapy. The patient received a total of two cycles of chemotherapy. Unfortunately, he passed away four months after his initial presentation due to complications of chemotherapy - neutropenic fever and septic shock - and worsening malignancy.

## Discussion

Ewing sarcoma is a malignant, small, round, blue-cell tumor of disputed origin that is one of the most aggressive types of sarcomas. It affects people of all ages but is predominantly seen in the pediatric population (median age at diagnosis: 15-16 years) [4,5]. Approximately 76.3% of Ewing sarcoma patients are between the ages of 0 to 24 years [2]. An age-adjusted analysis of the incidence of Ewing sarcoma revealed the highest rate in patients between 10 years and 19 years [2]. It is typically seen arising from bones but can present as an extraosseous tumor in rare cases [2]. 

Ewing sarcoma has an overall incidence of one case per million cancer diagnoses per year in the United States. Extraosseous Ewing sarcoma represents anywhere from 16 to 31% of these cases and has an estimated incidence rate of 0.4 per million [2,5,6]. The true incidence is probably underestimated, as it may occasionally be misdiagnosed as small cell carcinoma due to their similar morphology and occasional expression of neuroendocrine markers such as synaptophysin and chromogranin A [7]. Common locations associated with this condition are the chest wall, paravertebral area, pelvis and hip, and extremities [8].

Retroperitoneal Ewing sarcoma is a rare subtype of extraosseous Ewing sarcoma, and it has been reported in a limited number of case studies. It typically affects men in their second or third decades of life and has a high incidence of recurrence and metastasis [9]. The clinical presentation depends on the tumor size, location, depth of invasion, and growth speed. These rapidly growing masses often present insidiously due to the capacity of retroperitoneal space to accommodate the tumors, thereby delaying diagnosis [10]. By the time of detection, tumors may be large and necrotic. Patients often present with nonspecific symptoms associated with compression and mass effects, such as abdominal discomfort, intermittent pain, or a palpable mass [1]. The ambiguity of the clinical presentation, along with the tumor’s aggressive malignant potential, makes clinical suspicion critical for early diagnosis. 

Ewing sarcoma and retroperitoneal Ewing sarcoma are driven by specific chromosomal translocations, most commonly t(11;22)(q24;q12) and t(21;22)(q22;q12), which account for approximately 70-80% of all Ewing sarcoma cases [9]. This translocation results in the fusion of the EWSR1 gene (chromosome 22) with the FLI1 gene (chromosome 11). The resulting fusion protein acts as a transcription factor and drives enhanced oncogenesis. This FET-ETS fusion between a member of the FET family of genes, usually EWSR1 or FUS, and a member of the ETS (E26 transformation-specific or erythroblast transformation-specific) family of transcription factors, such as FLI1 or ERG, is a marker of Ewing sarcoma [7]. Other fusion partners seen with Ewing sarcoma include FEV, ETV1, and ETV4 [11].

The most specific immunohistological marker used in diagnosing retroperitoneal Ewing sarcoma is NKX2.2, while CD99 and FLI1 may serve as supporting markers in confirming the diagnosis [12]. Additionally, nuclear PAX7 positivity is expressed in a majority of Ewing sarcomas [11]. The combined use of these markers enhances diagnostic specificity, but they are not universally expressed in all cases. To minimize diagnostic uncertainty, FISH analysis is commonly utilized to detect EWSR1 gene rearrangements [12]. However, a negative FISH analysis does not exclude a diagnosis of Ewing sarcoma as the fusion protein may include FUS, a cryptic ESWR1, or ESWR1-ERG [11]. If necessary, next-generation RNA sequencing, which is highly sensitive and specific, can be utilized to identify the fusion partner for diagnosis [11]. Per the WHO diagnosis criteria, the essential diagnosis of Ewing sarcoma is based on a combination of small round cell morphology and CD99 membranous expression, but it is also desirable to detect FET-ETS fusions [7].

Since only a few cases of retroperitoneal Ewing sarcoma have been reported in the literature so far, standard guidelines for its treatment have not been established. Often, the recommended primary treatment is surgical resection to completely remove the tumor while achieving negative margins to lower the risk of recurrence and metastasis. This procedure presents significant challenges due to the tumor's proximity to critical structures in the retroperitoneum, such as the iliopsoas muscle, adrenal glands, and vascular structures. A multimodal approach involving chemotherapy and radiotherapy should be considered if complete resection is not feasible. 

High-dose chemotherapy, targeted therapy, and immunotherapy play a vital role in treatment [1]. Treatment guidelines include dose-intensive chemotherapy with VDC/IE (vindesine, adriamycin, cyclophosphamide, ifosfamide, and etoposide), VIDE (vincristine, adriamycin, isocyclophosphamide, and etoposide), and VAI (vincristine, actinomycin D, and isocyclophosphamide) [9]. A literature review of 13 cases reported that the VDC/IE combination led to significant tumor shrinkage in two young patients after six cycles of treatment. Anti-angiogenic drugs that target VEGF (vascular endothelial growth factor) or VEGF receptors have shown efficacy in 13 cases of primary retroperitoneal Ewing sarcoma, resulting in tumor shrinkage, no progression in the tumor growth, and, in one case, complete remission [9]. Even after surgical and chemotherapy treatment, patients have a poor prognosis of 10%, often with recurrence or metastasis [10,13]. The survival rate is 70.3% at two years, 49.7% at five years, and 41.9% at 10 years [9]. Our patient presented with necrosis, which may be a favorable prognostic factor correlated with a higher survival rate [14].

## Conclusions

This report highlights the importance of further research into extraosseous Ewing sarcoma due to its rapid-growing but slow-presenting nature that can quickly progress into an aggressive tumor. Retroperitoneal Ewing sarcoma is a rare subtype with limited data in the literature, and it can masquerade as other common abdominal and flank pathologies. It should still be considered in the differential diagnosis despite this tumor possibly occurring at younger ages, as seen in our patient. When working up retroperitoneal masses, taking a core needle biopsy and including the appropriate immunohistological panel and FISH analysis for Ewing sarcoma to consider retroperitoneal Ewing sarcoma is essential. While advances in chemotherapy and surgical techniques have greatly improved outcomes for patients with Ewing sarcoma, retroperitoneal Ewing sarcoma continues to have poor prognoses and low survival rates. Further research is necessary to better understand the pathophysiological causes of retroperitoneal Ewing sarcoma and optimize treatment protocols.
